# Dynamic Risk Assessment for the Development of Persistent Atrial Fibrillation Using Statistical and Machine Learning Approaches

**DOI:** 10.1111/anec.70229

**Published:** 2026-08-09

**Authors:** Alexei Nakonechnyi, Shaul Geliaks, Ilan Goldenberg, Dillon J. Dzikowicz, Emre Aktas, Susan Schleede, Scott McNitt, Agam Purohit, Burr W. Hall, Spencer Z. Rosero, David Z. Huang, Amole O. Ojo, Wojciech Zareba, Michael Christof, Mehmet K. Aktas

**Affiliations:** ^1^ University of Rochester Medical Center Rochester New York USA

**Keywords:** atrial fibrillation, cardiac implantable electronic devices, disease progression, machine learning, prediction model, risk assessment

## Abstract

**Background:**

Cardiac implantable electronic devices (CIEDs) frequently detect brief, often subclinical atrial fibrillation (AF), but their value for predicting progression to persistent AF remains uncertain. Statistical and machine learning (ML) approaches may enable dynamic risk stratification using this longitudinal device data.

**Objective:**

To develop a risk stratification model using clinical and CIED‐derived AF burden measured over a rolling 6‐month window to predict progression to persistent AF.

**Methods:**

We analyzed continuous CIED data from 1985 patients without prior persistent AF implanted between 2016 and 2024 at a tertiary medical center. AF burden and clinical variables were summarized using overlapping 6‐month rolling windows to estimate 1‐year risk of persistent AF. Associations were evaluated using Kaplan–Meier and Cox proportional hazards models. A gradient‐boosted decision tree model (XGBoost) was used to predict progression.

**Results:**

During a mean follow‐up of 1192 days, 874 patients (44%) developed paroxysmal AF, of whom 257 (29%) progressed to persistent AF after a mean of 813 days. Patients with no AF or < 1 h/day of AF in the prior 6 months had > 97% 1‐year freedom from persistent AF, whereas those with > 8 h/day had a 63% progression rate. Higher AF burden was strongly associated with progression (maximum HR 8.66, *p* < 0.001). The ML model demonstrated high predictive performance (sensitivity 99.4%, specificity 95.7%).

**Conclusion:**

CIED‐detected AF burden is strongly associated with progression to persistent AF. ML‐based analysis of 6‐month device data enables accurate, point‐in‐time risk stratification to support earlier and more targeted clinical management.

## Graphical abstract

Machine learning using 6‐month rolling AF burden from cardiac implantable devices accurately predicts 1‐year progression to persistent atrial fibrillation. Higher AF intensity and frequency strongly identify high‐risk patients, enabling dynamic, point‐in‐time risk stratification to support earlier, targeted clinical intervention.

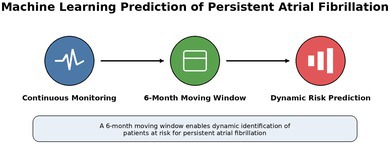

## 1 Introduction

Atrial fibrillation (AF), the most prevalent sustained cardiac arrhythmia, is a significant and escalating global health concern (Linz et al. 2024). In 2019, the worldwide prevalence of AF was estimated at 59.7 million cases, reflecting a substantial increase from previous decades (Linz et al. 2024). Projections indicate a continued upward trend, with cases in the Americas expected to reach 15.9 million by 2050 and 17.9 million in Europe by 2060 (Linz et al. 2024). The lifetime risk of developing AF is estimated to be between one in three to one in five individuals after the age of 45 (Vinter et al. 2024). AF is associated with serious adverse outcomes, including a fivefold increase in stroke risk, a twofold increase in myocardial infarction risk, and heightened susceptibility to dementia and cognitive decline (Ogawa et al. 2018). Notably, the risk of these outcomes varies among AF subtypes, with patients having more persistent and permanent forms of AF facing the highest risk (Ogawa et al. 2018; Noseworthy et al. 2019).

The proliferation of cardiac implantable electronic devices (CIEDs) has facilitated a more nuanced understanding of AF subtypes. CIEDs, such as pacemakers, implantable cardioverter defibrillators, and implantable cardiac monitors, offer continuous, long‐term cardiac rhythm monitoring, enabling the detection of brief, often subclinical AF episodes and allowing quantification of AF burden (Noseworthy et al. 2019). Previous studies have not utilized data from CIEDs to make a risk prediction for when a patient may transition from having paroxysmal to persistent AF. Machine learning (ML) algorithms excel at identifying subtle patterns and trends in AF that may elude human analysis. This study integrates traditional statistical approaches with ML to achieve dynamic risk stratification through a rolling window approach, evaluating the relationship between AF burden over six months and progression to persistent AF within one year. By leveraging longitudinal CIED data, this method provides real‐time, patient‐specific predictions. Our findings aim to refine clinical decision‐making and enhance targeted interventions, with future applications extending to emerging noninvasive continuous monitoring technologies such as wearables and patch‐based sensors, ultimately improving outcomes for clinicians and patients navigating the complexities of AF.

## 2 Methods

### 2.1 Study Design and Population

This study cohort included 1985 patients who received a CIED between 2016 and 2024 at the University of Rochester Medical Center (Rochester, NY, USA). Only patients implanted with devices capable of recording daily AF burden—including pacemakers, implantable loop recorders, implantable cardioverter‐defibrillators (ICDs), and cardiac resynchronization therapy defibrillators (CRT‐Ds)—were eligible for inclusion. Patients with a prior diagnosis of persistent AF at the time of implantation were excluded. Demographic characteristics, clinical history, and medication use were extracted from the electronic health record beginning at the time of device implantation. CIED interrogation data, including in‐office and remotely transmitted data, were longitudinally collected. The study was approved by the University of Rochester Institutional Review Board, and all data were managed in accordance with institutional protocols for patient privacy and data security.

### 2.2 URMC CIED Database

Patient data were obtained from the University of Rochester Medical Center (URMC) CIED Database, which integrates structured electronic medical record (EMR) data with device interrogation records. Clinical data were collected between January 1, 2016, and January 1, 2024. Follow‐up began at the time of CIED implantation and continued throughout the study duration. The study endpoint—development of persistent AF—was defined as continuous AF lasting more than 7 consecutive days with ≥ 24 h of AF recorded each day, as documented in CIED logs. Daily AF burden was measured as the cumulative duration of AF episodes per day and summarized over a rolling six‐month window for each patient. For each window, we calculated the average AF duration on AF‐active days and the proportion of days with any AF (AF‐active days) as distinct burden metrics. Demographic and clinical characteristics were extracted from the EMR and matched to CIED‐derived AF data to support baseline comparisons between patients with paroxysmal AF and those who progressed to persistent AF.

### 2.3 Statistical Analysis and ML Modeling

To evaluate the relationship between AF burden and development of persistent AF, we applied both survival analysis and ML–based risk prediction. A 6‐month rolling window was applied at 24‐h intervals across the follow‐up period to dynamically stratify patients by AF burden and estimate 1‐year risk of persistent AF. This approach generated multiple overlapping observations per patient over time. Patients were followed from the time of CIED implantation for up to one year. Censoring occurred upon the development of persistent AF, death, or loss to follow‐up. Kaplan–Meier methods were used to estimate time to progression, and univariate Cox proportional hazards models were used to assess associations between AF burden and the risk of persistent AF. Since Kaplan–Meier estimation requires fixed exposure groups at baseline, we implemented a landmark design in which the landmark marks the start of each follow‐up window. At each landmark, AF‐burden category was defined using data from the preceding window (e.g., 6 months before the landmark) and then held fixed for the subsequent survival period. Patients who had already reached persistent AF before a given landmark were excluded from that window's risk set. Survival time was measured from the landmark forward. All statistical tests, including log‐rank comparisons for Kaplan–Meier curves and Cox regression analyses, were two‐sided, with a significance threshold of *p* < 0.05. As a secondary analysis, we assessed whether the association between AF burden and progression to persistent AF was independent of underlying cardiac function. Among patients with available left ventricular ejection fraction (LVEF) data, we fit Cox proportional hazards models adjusted for LVEF as a continuous covariate. These adjusted models were constructed using the same AF burden groups and outcomes as the primary univariate analysis, and results were compared to the unadjusted hazard ratios to assess consistency in risk estimates and relative rankings. We additionally fit a multivariable Cox proportional hazards model adjusting for demographic and clinical covariates, including age, sex, LVEF, NYHA functional class, and use of beta‐blockers, angiotensin‐converting enzyme inhibitors (ACEi), angiotensin receptor blockers (ARB), angiotensin receptor–neprilysin inhibitors (ARNI), and sodium–glucose cotransporter‐2 inhibitors (SGLT2i).

To complement association‐based models, we developed a predictive framework using gradient‐boosted decision trees (XGBoost). The model was trained using features derived from the prior six months, including average AF duration on AF‐active days, the proportion of AF‐active days, demographic characteristics, and medication use. At each 24‐h time point, the model used the preceding 6 months of data to predict the risk of persistent AF within the following year. Overlapping time windows from the same patient were treated as independent samples. Model performance was evaluated using sensitivity, specificity, F1‐score, confusion matrices, and area under the receiver operating characteristic curve (AUC‐ROC). Internal validation was conducted using a holdout test set. Feature importance was assessed using XGBoost's gain‐based metric to support interpretability. All statistical and machine learning analyses were performed using Python (version 3.12) with the scikit‐learn and XGBoost libraries.

## 3 Results

### 3.1 Patient Characteristics and AF Progression Rates

Of the 1985 patients included in the cohort, 874 (44%) developed AF during a mean follow‐up of 1192 days. Among those, 617 patients (71%) remained in paroxysmal AF, while 257 patients (29%) progressed to persistent AF after a mean of 813 days. The mean age of the overall cohort was 70 ± 16 years, with 60% male and 87% identifying as White. Patients who progressed to persistent AF were older (76 ± 11 years), had lower LVEF (46% ± 11% vs. 54.9% ± 12%), and were more likely to have higher NYHA functional class (III/IV: 11%) compared to those who remained in paroxysmal AF or those with no AF at any time during follow‐up (Table 1). Differences in race and ethnicity were minimal across groups, but patients who developed persistent AF were more likely to be on beta‐blockers (93%), anticoagulants (99%), and diuretics (89%) than those with paroxysmal AF or no AF (Table 1).

**TABLE 1:** Population clinical demographic and clinical characteristics.

Parameter	No AF (*N* = 1111)	Paroxysmal AF (*N* = 617)	Persistent AF (*N* = 257)	All (*N* = 1985)	*p*
Age (average)	68.99 (*N* = 1111)	71.0 (*N* = 617)	76.53 (*N* = 257)	70.59 (*N* = 1985)	< 0.001
Male	647 (58.24%)	391 (63.37%)	156 (60.7%)	1194 (60.15%)	0.111
NYHA Class I	21 (1.89%)	10 (1.62%)	5 (1.95%)	36 (1.81%)	0.909
NYHA Class II	62 (5.58%)	30 (4.86%)	31 (12.06%)	123 (6.2%)	< 0.001
NYHA Class III	101 (9.09%)	38 (6.16%)	28 (10.89%)	167 (8.41%)	0.034
NYHA Class IV	34 (3.06%)	29 (4.7%)	15 (5.84%)	78 (3.93%)	0.059
NYHA Class Unknown	893 (80.38%)	510 (82.66%)	178 (69.26%)	1581 (79.65%)	< 0.001
LVEF (average)	54.90 (*N* = 660)	49.66 (*N* = 332)	46.47 (*N* = 127)	47.31 (*N* = 1119)	< 0.001
White or Caucasian	946 (85.15%)	544 (88.17%)	238 (92.61%)	1728 (87.05%)	0.004
Black or African American	106 (9.54%)	50 (8.1%)	13 (5.06%)	169 (8.51%)	0.061
Hispanic or Latino	27 (2.43%)	11 (1.78%)	4 (1.56%)	42 (2.12%)	0.536
Other	59 (5.31%)	23 (3.73%)	6 (2.33%)	88 (4.43%)	0.067
Beta Blocker	948 (85.33%)	548 (88.82%)	238 (92.61%)	1734 (87.36%)	0.003
Anticoagulation	891 (80.20%)	573 (92.87%)	254 (98.83%)	1718 (86.55%)	< 0.001
Diuretic	836 (75.25%)	491 (79.58%)	229 (89.11%)	1556 (78.39%)	< 0.001
Statin	885 (79.66%)	499 (80.88%)	231 (89.88%)	1615 (81.36%)	< 0.001
Calcium Channel Blocker	534 (48.06%)	336 (54.46%)	159 (61.87%)	1029 (51.84%)	< 0.001
ACEi/ARB/ARNI	762 (68.59%)	414 (67.1%)	183 (71.21%)	1359 (68.46%)	0.488

*Note:* The *p*‐value represents comparison across the three AF groups (No AF, Paroxysmal AF, and Persistent AF).

Abbreviations: ACEi, angiotensin‐converting enzyme inhibitor; ARB, angiotensin receptor blocker; ARNI, angiotensin receptor–neprilysin inhibitor; NYHA, New York Heart Association.

### 3.2 Kaplan–Meier Estimates of Persistent AF Risk

**FIGURE 1:**
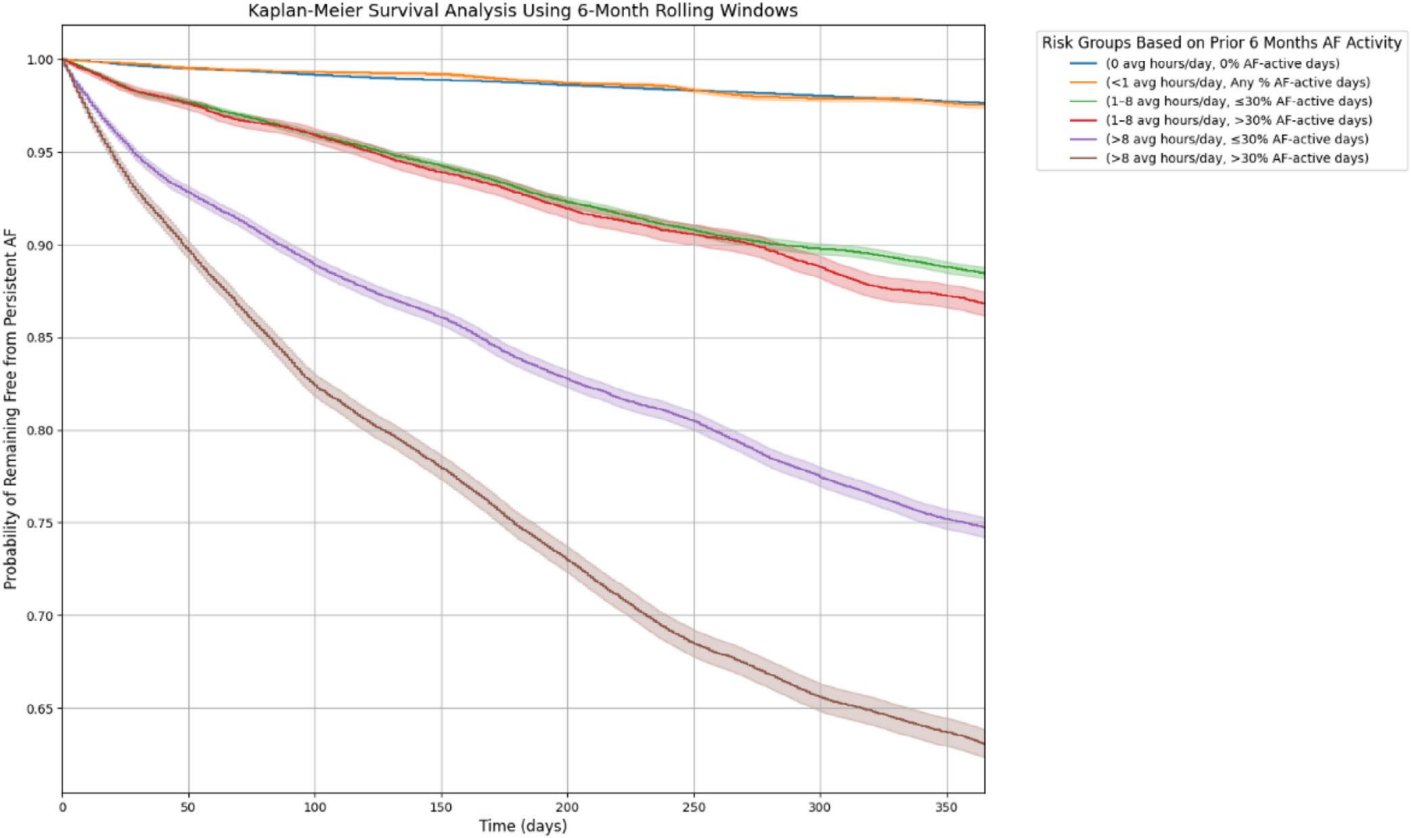
Survival analysis. Kaplan–Meier curves by AF burden category.

Kaplan–Meier analysis showed that risk of developing persistent AF increased substantially with higher AF burden: those with > 8 h/day and AF present on > 30% of days had a 63% probability of remaining persistent AF–free at one year. In contrast, patients with no AF in the preceding six months had a 98% probability of remaining free from persistent AF at one year, and patients with < 1 h/day of AF burden averaged over any percentage of days had a 97% probability of remaining free from persistent AF (Figure 1).

### 3.3 Cox Proportional Hazard Model Association Between AF Burden and Persistent AF Risk

Cox proportional hazards models confirmed that higher AF burden over the prior six months was significantly associated with the development of persistent AF, relative to patients with no AF during that period. Compared to patients with no AF in the prior six months, those with < 1 h/day of AF had a modest 13% statistically significant increase in risk of developing persistent AF. In contrast, risk increased markedly in all higher AF burden groups. Patients with 1–8 h/day of AF—regardless of whether it occurred on < 30% or > 30% of days—had similar > 4‐fold elevated risks of developing persistent AF (HRs: 4.42 and 4.41, respectively). The risk of developing persistent AF was even higher (> 6‐fold) among those with > 8 h of AF/day on < 30% of days (HR: 6.32), and highest (> 8‐fold) in those with > 8 h of AF/day on > 30% of days (HR: 8.66) (Table 2).

**TABLE 2:** Association between AF burden and risk of progression to persistent AF (reference: no AF in prior 6 months).

AF burden group	Hazard ratio (HR)	Coefficient (*β*)	*p*
< 1 h/day, any % AF‐active days	1.13	0.1233	0.0002
1–8 h/day, < 30% AF‐active days	4.42	1.4861	< 0.0001
1–8 h/day, > 30% AF‐active days	4.41	1.4834	< 0.0001
> 8 h/day, < 30% AF‐active days	6.32	1.8437	< 0.0001
> 8 h/day, > 30% AF‐active days	8.66	2.1585	< 0.0001

### 3.4 Multivariate Modeling Adjusting for Demographic, Clinical, Treatment and Imaging Variables

To further assess the robustness of our findings, we conducted a multivariable Cox proportional hazards analysis adjusting for age, sex, NYHA functional class (or diuretic use), LVEF, and key cardiovascular medications (beta blockers, ACEi/ARB/ARNI, and SGLT2 inhibitors). The results remained consistent, with > 8 h of AF/day on > 30% of days associated with the highest risk of progression to persistent AF (HR: 2.01, *p* < 0.0001), and moderate AF burden (1–8 h of AF/day) also significantly increasing risk regardless of AF frequency (Table 3). Notably, the relative ordering of AF burden groups by risk remained unchanged compared to both the unadjusted and LVEF‐adjusted models, reinforcing the independent and graded relationship between AF burden and progression to persistent AF.

**TABLE 3:** Multivariable association between AF burden and risk of progression to persistent AF adjusted for clinical covariates (reference: no AF in prior 6 months).

Predictor	Hazard ratio (HR)	Coefficient (*β*)	*p*
< 1 h/day, any % AF‐active days	0.98	−0.0240	0.1328
1–8 h/day, ≤ 30% AF‐active days	1.14	0.1293	0.0002
1–8 h/day, > 30% AF‐active days	1.16	0.1478	0.0542
> 8 h/day, ≤ 30% AF‐active days	1.44	0.3639	< 0.0001
> 8 h/day, > 30% AF‐active days	2.01	0.7004	< 0.0001
Left ventricular ejection fraction (LVEF)	1.00	−0.0005	0.3341
Age	1.00	0.0040	< 0.0001
Male sex (ref: female)	1.00	0.0045	0.7941
NYHA Class ≥ II or on diuretic	1.05	0.0461	0.0033
Beta blocker use	1.05	0.0481	0.0045
ACEi/ARB/ARNI use	0.99	−0.0059	0.6982
SGLT2 inhibitor use	1.02	0.0215	0.3262

### 3.5 Machine Learning Prediction of Persistent AF

**FIGURE 2:**
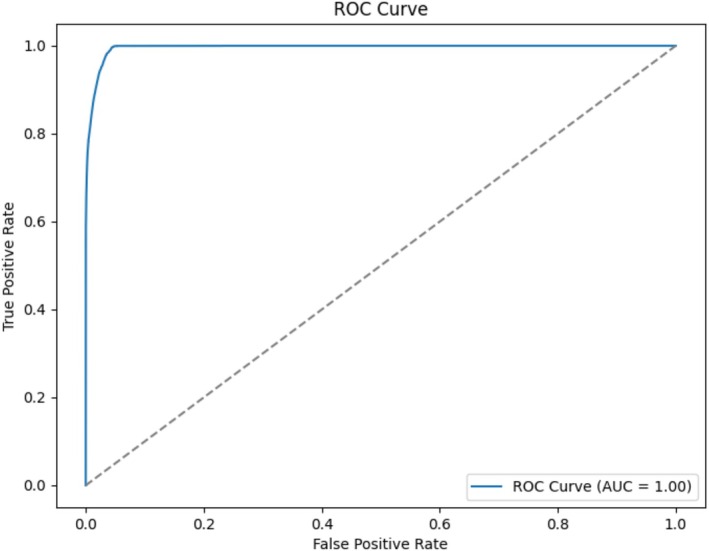
ROC curve analysis. Receiver operating characteristic (ROC) curve illustrating the model's AUC of 0.9957, demonstrating its discrimination ability between progressive and non‐progressive paroxysmal AF cases.

**FIGURE 3:**
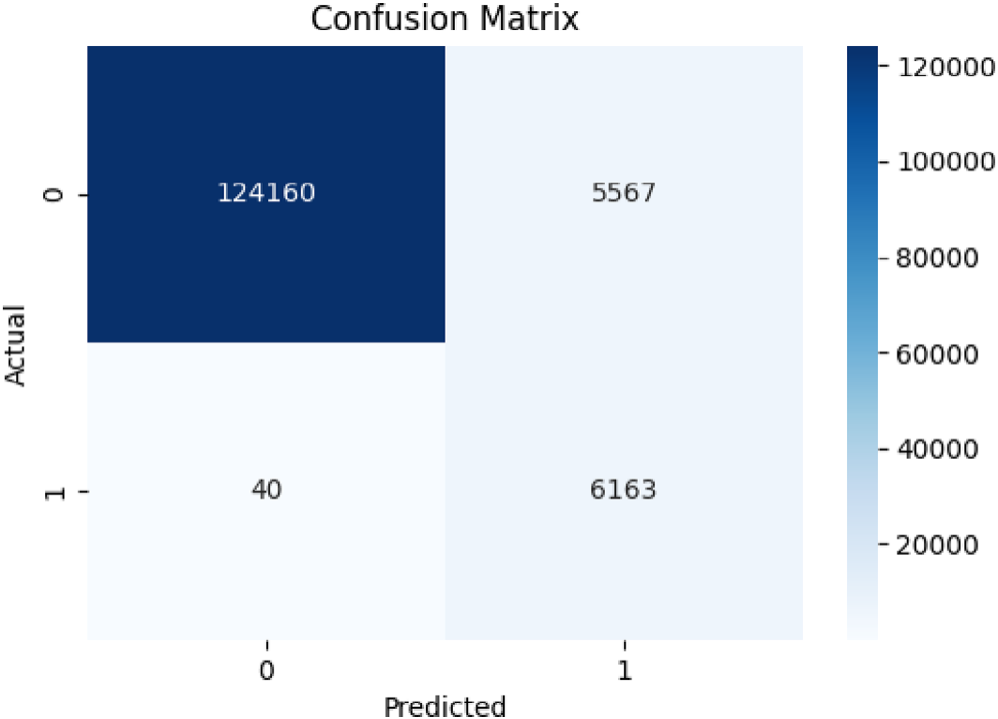
Confusion matrix. A detailed matrix showing true positives, true negatives, false positives, and false negatives for the machine learning model.

**FIGURE 4:**
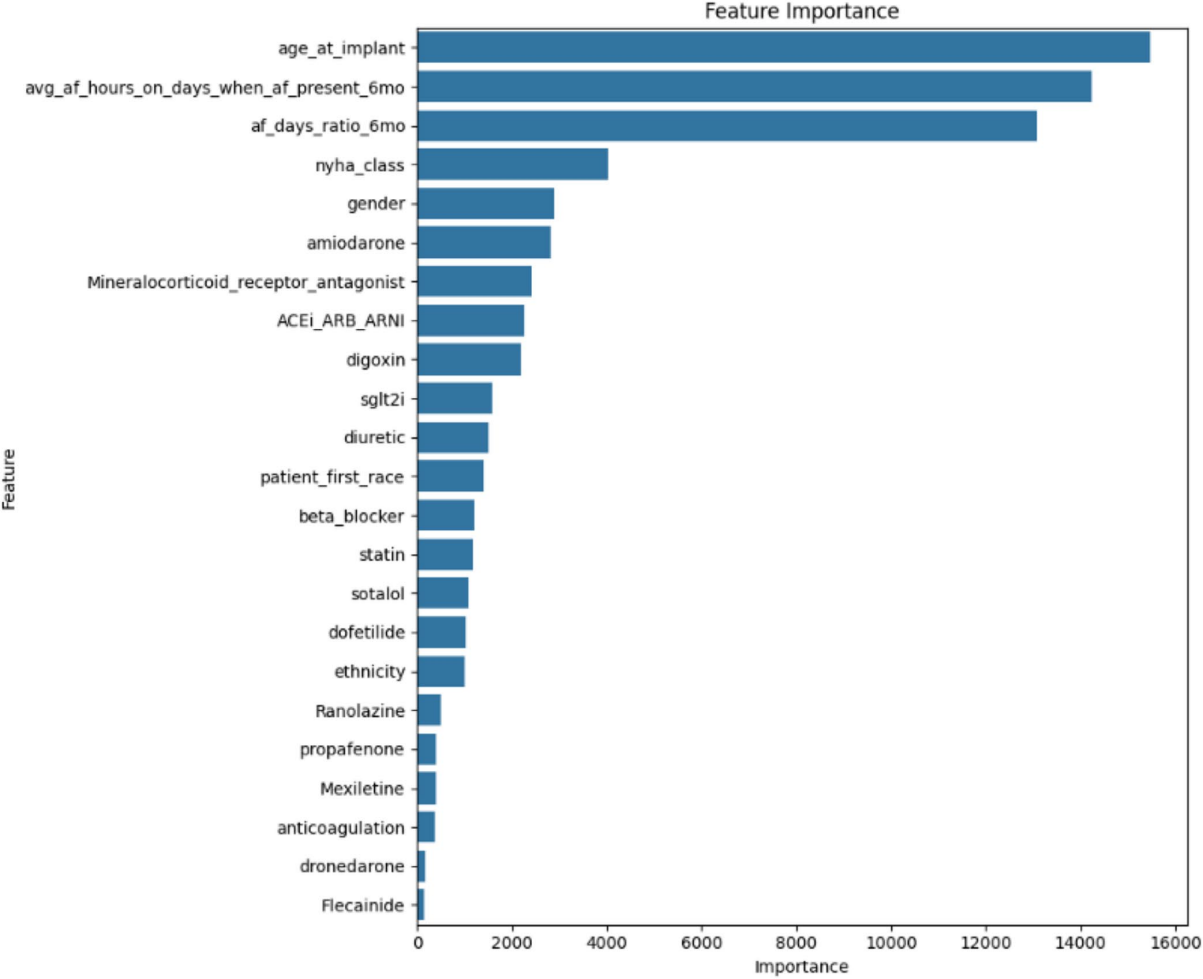
Feature importance. Visualization of the relative importance of predictors used by the machine learning model (top features like age at implant, the average duration of AF episodes on AF‐active days over the prior six months, the ratio of AF‐active days to total days, NYHA class, and others).

Our machine learning model demonstrated excellent predictive performance for identifying patients at risk of developing persistent AF using data from the preceding six months. The model achieved an area under the receiver operating characteristic curve (AUC‐ROC) of 0.996, indicating great discrimination (Figure 2). The confusion matrix (Figure 3) showed 124,160 true negatives and 6163 true positives, with only 40 false negatives and 5567 false positives, reflecting high sensitivity and strong overall discriminative performance, though with a modest false positive rate. Feature importance analysis revealed that the most informative predictors included average AF duration on AF‐active days, the proportion of AF‐active days, and age at implant (Figure 4). NYHA functional class and sex also contributed to model performance, while medication use—though retained in the model—had lower relative importance (Figure 4).

## 4 Discussion

Our study demonstrates that machine learning models grounded in longitudinal CIED data can accurately predict the development of persistent AF within one year. These findings suggest that leveraging a six‐month rolling window approach enables precise risk stratification and provides new insights into AF progression dynamics, which are currently not captured in conventional risk models.

### 4.1 CIED‐Detected Time‐Dependent Risk Factors for the Development of Persistent AF

The most influential predictors in our model included age at implant, AF burden on AF‐present days, and the proportion of AF‐active days over six months, highlighting the strong association between AF burden and disease progression. These findings align with previous studies demonstrating that higher AF burden is associated with an increased risk of stroke, heart failure, and long‐term adverse remodeling leading to heart failure (Noseworthy et al. 2019; Wong et al. 2018; Steinberg et al. 2015; Tanaka et al. 2020; Healey et al. 2012). The ASSERT trial previously reported that AF episodes lasting longer than 6 min were associated with a significantly higher risk of ischemic stroke, emphasizing the clinical importance of early detection and burden quantification (Healey et al. 2012). Furthermore, the AF burden as detected by CIEDs has been shown to predict stroke risk more effectively than traditional CHA_2_DS_2_‐VASc scores alone, underscoring the importance of continuous monitoring (Tiver et al. 2021).

### 4.2 Clinical Risk Factors for the Development of Persistent AF

Another significant finding was the role of NYHA functional class and sex in predicting AF progression. Heart failure severity, as indicated by NYHA functional class III–IV symptoms, was the fourth most significant predictor of AF progression, consistent with prior research suggesting that heart failure contributes to adverse atrial remodeling and AF persistence (Santhanakrishnan et al. 2016). Additionally, female patients exhibited a higher likelihood of AF progression, which aligns with existing literature showing that women with AF have a higher thromboembolic risk and may experience faster progression to persistent forms of the disease (Ko et al. 2017). However, additional studies are needed to explore potential sex‐specific pathophysiological mechanisms underlying AF progression.

### 4.3 Potential Clinical Implications

Our findings suggest that machine learning applied to continuous CIED data can accurately predict the development of persistent AF, addressing a critical gap in AF management. Identifying patients at risk of progression is particularly important because once AF becomes persistent, available therapies, including catheter ablation, are significantly less effective (Gunawardene and Willems 2022; De Vos et al. 2010). Prior studies have demonstrated that early ablation can modify disease progression and improve long‐term outcomes, supporting a proactive approach to rhythm control before AF becomes refractory (Kuck et al. 2021; Andrade et al. 2021). As catheter ablation is less effective once AF becomes persistent, early identification of high‐risk patients may improve the timing and success of rhythm‐control strategies. Our results may be incorporated into risk stratification paradigms that integrate AF burden dynamics to better guide treatment timing, particularly in selecting candidates for early catheter ablation. Our data suggest that both AF intensity (AF hours/day) and AF frequency (AF‐active days) independently contribute to progression risk, reinforcing the need to move beyond single‐threshold burden definitions. Given that AF progression is driven by complex structural and electrical remodeling (Gunawardene and Willems 2022; De Vos et al. 2010), a predictive model capable of detecting these early changes could enable intervention before irreversible atrial remodeling occurs, thereby preserving long‐term treatment efficacy. Future studies should assess whether incorporating machine learning‐driven risk stratification into clinical workflows can improve patient outcomes by optimizing the timing of rhythm‐control strategies and preventing the transition to persistent AF.

Looking ahead, these insights may be adaptable to emerging forms of noninvasive continuous monitoring. Wearables, patch‐based ECG systems, and other ambulatory sensors now generate longitudinal rhythm data that resemble many features captured by CIEDs. As these technologies mature, similar machine learning models could be applied to noninvasive datasets to identify early patterns of AF burden associated with progression. Extending this framework to wearable and patch‐derived signals may broaden opportunities for population‐level screening and earlier recognition of patients at elevated risk, particularly among those without implanted devices. Incorporating these approaches into future monitoring strategies could support a more accessible and proactive model of AF management, complementing device‐based risk assessment and further refining the timing of rhythm‐control interventions.

Importantly, our study demonstrated strong concordance between conventional statistical methods and ML‐based prediction. The Cox proportional hazards models and the ML model identified overlapping high‐risk features—AF intensity (AF hours/day) and AF frequency (AF‐active days)—underscoring the robustness and generalizability of these predictors. The hazard ratios across burden categories were consistent after further adjustment for demographic, clinical, imaging and treatment variables, and the relative ranking of risk was preserved. These findings reinforce the conclusion that AF burden—captured through both intensity and frequency—is an independent and reliable marker of disease progression, even after accounting for underlying cardiac function. The alignment between conventional statistical methods and machine learning approaches strengthens confidence in the clinical relevance of these variables and suggests that ML can enhance, rather than replace, traditional risk assessment methods. The consistency between these approaches supports a unified framework for dynamic risk stratification that leverages both interpretability and predictive power.

### 4.4 Limitations

Our study has several limitations. First, this was a single‐center retrospective analysis, which may limit generalizability. Future multicenter validation studies are necessary to confirm the robustness of our findings across diverse populations. Second, while CIEDs provide continuous monitoring, they are not universally available, particularly in low‐resource settings. As wearable technology advances, future studies should explore whether non‐invasive wearables, such as smartwatches with photoplethysmography and electrocardiographic capabilities, can provide similar predictive value in AF burden assessment. Additionally, our study did not evaluate the impact of early interventions on AF progression. Future research should investigate whether targeted rhythm‐control interventions, guided by machine learning risk stratification, can alter disease trajectory and reduce cardiovascular complications^16^. Because AF burden was calculated over rolling 6‐month windows, multiple observations per patient may violate the assumption of independence. We acknowledge that repeated measures introduce correlation, and future work should explore frailty models or generalized estimating equations. Prospective randomized trials would be essential to validate the clinical utility of machine learning‐driven risk stratification in real‐world AF management.

## 5 Conclusion

This study demonstrates that machine learning models leveraging longitudinal CIED data can accurately predict the development of persistent AF. Using a six‐month rolling window of AF burden, both traditional statistical analyses and machine learning methods independently identified similar high‐risk phenotypes, including AF intensity (AF hours/day) and AF frequency (AF‐active days). This convergence reinforces the validity of our findings and supports the integration of dynamic AF burden metrics into clinical risk stratification. Incorporating these predictive tools into practice may improve the timing of rhythm‐control strategies and prevent irreversible atrial remodeling. Future multicenter studies and prospective trials are warranted to validate these insights and assess their impact on outcomes in real‐world AF management. As noninvasive monitoring technologies mature, these methods may also be extended to data obtained from wearable and patch‐based sensors. Applying similar machine learning frameworks to longitudinal rhythm data collected outside of implanted devices could broaden opportunities for early identification of patients at risk for progression. Incorporating noninvasive monitoring into future validation efforts will clarify how these approaches can complement device‐based strategies and support a more accessible, proactive model of AF management.

## Author Contributions

Alexei Nakonechnyi, Shaul Geliaks, Ilan Goldenberg, Dillon Dzikowicz, Emre Aktas, Susan Schleede, Scott McNitt, Agam Purohit, Burr Hall, Spencer Rosero, David Huang, Amole Ojo, Wojciech Zareba, Michael Christof, and Mehmet Aktas made substantial contributions to the study and interpretation of its findings, participated in drafting or critically revising the manuscript, approved the final version, and agree to be accountable for the work.

## Funding

The authors have nothing to report.

## Ethics Statement

The study was approved by the University of Rochester Institutional Review Board, and all data were managed in accordance with institutional protocols for patient privacy and data security.

## Conflicts of Interest

The authors declare no conflicts of interest.

## Data Availability

The data that support the findings of this study are available from the corresponding author upon reasonable request.
